# Silent voices: Family caregivers' narratives of involvement in palliative care

**DOI:** 10.1002/nop2.344

**Published:** 2019-08-10

**Authors:** Anett Skorpen Tarberg, Marit Kvangarsnes, Torstein Hole, Morten Thronæs, Torfinn Støve Madssen, Bodil J. Landstad

**Affiliations:** ^1^ Helse Møre og Romsdal Hospital Trust Ålesund Norway; ^2^ European Palliative Care Research Centre (PRC), Department of Clinical and Molecular Medicine, Faculty of Medicine and Health Sciences Norwegian University of Science and Technology (NTNU) Trondheim Norway; ^3^ Faculty of Medicine and Health Sciences, Institute of Health Sciences Ålesund Norwegian University of Science and Technology (NTNU) Ålesund Norway; ^4^ Department of Circulation and Medical Imaging, Faculty of Medicine and Health Sciences Norwegian University of Science and Technology (NTNU) Trondheim Norway; ^5^ Cancer Clinic, St. Olav Hospital Trondheim University Hospital Trondheim Norway; ^6^ Department of Health Sciences Mid Sweden University Östersund Sweden; ^7^ Levanger Hospital Nord‐Trøndelag Hospital Trust Levanger Norway

**Keywords:** cancer, caregivers, decision‐making, narratives, nurses, nursing, palliative care, primary health care

## Abstract

**Aim:**

To explore how family caregivers experience involvement in palliative care.

**Design:**

A qualitative design with a narrative approach was used.

**Methods:**

Purposive sampling and narrative interviews were conducted. Eleven bereaved family caregivers for patients with cancer receiving palliative care were interviewed in Mid‐Norway between November 2016–May 2017.

**Results:**

We identified four themes related to family caregivers' experiences of involvement in the early, middle, terminal and bereavement phases of palliative care: (a) limited involvement in the early phase; (b) emphasis on patient‐centred care in the middle phase; (c) lack of preparation for the dying phase; and (d) lack of systematic follow‐up after death. Family caregivers experienced low level of involvement throughout the palliative pathway.

**Conclusion:**

The involvement of family caregivers in palliative care may not be proportional to their responsibilities. The needs of family caregivers should be addressed in nursing education to give nurses competence to support family caregivers in providing home‐based care.

## 1 INTRODUCTION

Palliative care is an approach that improves the quality of life of patients facing life‐threatening illness and their families (World Health Organization, 2009). The European Association for Palliative Care (2008) has identified the following values for patients in hospice and palliative care in Europe (European Association for Palliative Care, 2008): autonomy, dignity, relationship between patient–healthcare professionals, quality of life, position towards life and death, communication, public education, multi‐professional and interdisciplinary approach and grief and bereavement. These values are mainly focused on the needs of the patient and conditions for providing good palliative care and the situation of family caregivers are less emphasized.

The symptom burden among patients in palliative care is often substantial (Laugsand, Kaasa, Hanks, & Klepstad, 2009; Teunissen et al., 2007). Home‐based palliative care is associated with improved symptom control, better quality of life for the patient and reduced use of healthcare resources (Rabow et al., 2013) and family caregivers play an essential role in facilitating home‐based palliative care (Knighting et al., 2016; Reigada, Pais‐Ribeiro, Novella, & Gonçalves, 2015; Woodman, Baillie, & Sivell, 2016). Internationally, there is a goal to promote home‐based care and it is therefore important to gain knowledge about how family caregivers experience involvement during different phases of palliative care.

### 1.1 Background

Home‐based palliative care entails more responsibility for family caregivers (Knighting et al., 2015; Proot et al., 2003). Several studies have shown that family caregivers balance the care burden with what they can cope with (Andershed, 2006; Proot et al., 2003; Woodman et al., 2016). According to one study, family caregivers who were supported in their role reported a positive home‐care experience, while some family caregivers felt pressure to provide home care from the patient, relatives or health professionals (Woodman et al., 2016). A study from Sweden showed that family caregivers could feel doubt, ambivalence and anxiety about providing palliative care. Family caregivers in that study did not consider the consequences of home‐based palliative care for themselves as long as it was the patient's clear wish (Linderholm & Friedrichsen, 2010). Care burden, restrictions on activities, fear, insecurity, loneliness, the prospect of death and lack of emotional, practical and information‐related support are factors that are considered to increase caregiver vulnerability and the risk for fatigue and burnout (Proot et al., 2003).

A meta‐synthesis (Fringer, Hechinger, & Schnepp, 2018) of studies from Europe, the United States and Canada showed that palliative patients and family caregivers wanted to maintain normality in their daily life. They experienced challenges dealing with their life situation and achieving balance in everyday life. Supportive and distressing factors influenced the patient and family caregivers when trying to maintain normality and they had to deal with changed roles when anticipating the future.

The dominant ideal in Western culture is individual autonomy, which emphasizes the patient's ability to make an informed, independent choice (Brogaard, Jensen, Sokolowski, Olesen, & Neergaard, 2011; Ho, 2008). This view is also supported in the recent Lancet Oncology Commission (Kaasa et al., 2018), which highlighted patient‐centred care. While several definitions of patient‐centred care exist, the concept generally refers to patient involvement in care and the individualization of patient care (Epstein & Street, 2011; Kitson, Marshall, Bassett, & Zeitz, 2013; Rathert, Wyrwich, & Boren, 2013). This understanding of patient‐centred care can be interpreted as contradictory to the World Health Organization's definition of palliative care (World Health Organization, 2009), which also emphasizes the needs of the family. Although some research has emphasized the family perspective in patient‐centred care, most research in palliative care has adopted the individual perspective (Etkind et al., 2015; Kitson et al., 2013; Rathert et al., 2013; Robinson, Callister, Berry, & Dearing, 2008). Professional care based on humanistic values may promote patients' and relatives' sense of coherence and involvement (Andershed & Ternestedt, 2001) and research has demonstrated that family caregivers who are involved in the decision‐making process cope better with home‐based care (Stajduhar & Davies, 2005). Jack, Mitchell, Cope, and O'Brien (2016) emphasized the importance of comprehensive care in supporting patients and older family caregivers.

Family caregivers may be considered an integrated component of the patient's identity and an important part of the patient's life (van Nistelrooij, Visse, Spekkink, & de Lange, 2017). Family caregivers are considered the key persons in palliative home care and may provide a holistic family view (Brogaard et al., 2011).

However, family caregivers who provide home‐based care may feel unprepared for the role and often neglect their own needs (Stajduhar & Davies, 2005). A study from Wales (Pottle, Hiscock, Neal, & Poolman, 2017) showed that while patients maintained a sense of normality by staying at home, family caregivers felt the opposite; their normality was lost. The patients' views and needs took precedence over those of the family caregivers. Family caregivers might experience more distress than the patient but receive less social support and some feel overwhelmed by the situation and the burden of making decisions without understanding the consequences of those decisions (Rakic et al., 2018). To be fully informed about how the disease is progressing and what could happen when the patient's disease becomes worse is seen as crucial (Knighting et al., 2015).

A study from Toronto (Mohammed et al., 2018) showed that the family caregivers felt they had to take a more active role when the patient received care at home. It was difficult to navigate in the home‐care system and to cooperate with all the different professional caregivers. Most of the family caregivers had never seen death before and they therefore needed detailed explanations about the dying process.

Palliative care involves bereavement support for family caregivers (World Health Organization, 2009), and assessment tools have been developed that evaluate family caregivers' need for support (Aoun, Bird, Kristjanson, & Currow, 2010; Ewing, Brundle, Payne, & Grande, 2013; Ewing & Grande, 2013; Knighting et al., 2015; Thomsen, Guldin, Nielsen, Ollars, & Jensen, 2017). Thomsen et al. (2017) demonstrated that 75% of family caregivers who underwent a systematic risk and need assessment received their own support plan, which enabled better follow‐up and more targeted support. The intervention was based on risk factors listed in the “Bereavement support standards for specialist palliative care services” (Hudson et al., 2012).

Røen et al. (2018) have explored factors promoting carer resilience. A personal relation to the healthcare providers was identified as a particularly important resilience factor. Available palliative care, information about the illness, prognosis and death were also important.

However, there is a lack of knowledge regarding family caregivers' experience of involvement in the different phases of the palliative care. Such knowledge may help to improve palliative care by identifying deficiencies in the different phases and this insight could be used to empower family caregivers and facilitate better care.

## 2 THE STUDY

### 2.1 Aim

The aim of the study was to explore how family caregivers experience involvement in palliative care. The research question was as follows: How do family caregivers experience information and involvement in the different phases of palliative care?

### 2.2 Design

This study had a qualitative design with a narrative approach (Chase, 2005; Holloway & Freshwater, 2007; Patton, 2015). A narrative approach was chosen to highlight the perspective of family caregivers (Holloway & Freshwater, 2007), and the narrative interviews were conducted with open‐ended questions (Brinkmann & Kvale, 2015).

Thompson's theoretical framework of five levels of patient‐desired involvement was used in the study (Thompson, 2007). Each level represents different positions of power, ranging from non‐involvement to full autonomy. Participation and involvement consist of the following five components: (a) contributing to action sequences; (b) influencing the problem definition; (c) sharing in the reasoning process; (d) influencing decision‐making; and (e) experiencing emotional reciprocity. Although the theory was developed to understand patient involvement, the components have been successfully applied to family caregivers and the theory therefore provides a useful framework for understanding family caregiver involvement (Aasen, Kvangarsnes, Wold, & Heggen, 2012).

### 2.3 Participants

The informants were chosen by purposive sampling of informants with maximum variation (Brinkmann & Kvale, 2015). The inclusion criteria were as follows: (a) the family caregivers had followed the patient closely in palliative care trajectory; (b) the patient had received services from both primary and specialist health care; (c) the family caregivers were able to speak the Norwegian language proficiently; (d) the family caregivers were older than 18 years; (e) the family caregivers had lost their relatives 3–12 months prior to the interview; and (f) cancer was the cause of death. We conducted eleven narrative interviews (Table 1).

**Table 1:** Characteristics of study participants

	Participants (total *N* = 11)
Interviewed in the relative's home	9
Interviewed in a community institution	2
Female	9
Male	2
Higher education	7
Lower education	3
Spouse	9
Daughter/son	2
<30 years	0
31–40 years	2
41–50 years	1
51–60 years	3
61–70 years	2
71–80 years	3

### 2.4 Data collection

The informants were recruited by oncology nurses in municipalities. The locations for the interviews were chosen by the family caregivers: nine participants were interviewed in their homes and two were interviewed in a public healthcare centre. The interviews were recorded and transcribed immediately afterwards (Creswell, 2014; Polit & Beck, 2012). The interviews were conducted by the first author. In the interviews, the open‐ended questions posed to the family caregivers focused on four pre‐defined phases of the palliative care: the early, middle, terminal and bereavement phases (Table 2). Prior to the interviews, the informants were informed of how the various phases were defined. The family caregivers were encouraged to lead the interviews. The interviewer had a passive role, supporting the interviewees (Holloway & Freshwater, 2007; Patton, 2015). The interviews took place between November 2016–May 2017 and lasted between 50–180 min. After 11 interviews, we considered the data to be saturated, as the data tended to become repetitive and redundant (Saunders et al., 2018).

**Table 2:** Interview guide

Can you tell me how you experienced the palliative care pathway?
How did you experience the information you received in different phases of the palliative care pathway (e.g., the early phase, middle phase, terminal phase, and bereavement phases)?
How did you experience being involved in the different phases of the palliative care pathway (e.g., the early phase, middle phase, terminal phase, and bereavement phases)?
Is there anything else you want to add?

### 2.5 Ethical considerations

The family caregivers could be grieving at the time of the interviews and this was taken into consideration during the interviews. The project was undertaken according to research ethics guidelines (General Assembly of the World Medical Association, 2014). Informed written consent was given by the participants at the start of the interview. The Regional Committee on Medical and Health Research Ethics determined that the study did not need approval (2016/978/REK NORD). The Data Protection Official for Research approved the study (2016/960‐25).

### 2.6 Data analysis

An inductive approach was adopted when analysing the interviews, with a focus on the narrative plot (Holloway & Freshwater, 2007). First, a holistic impression of the interviews was obtained (Brinkmann & Kvale, 2015). Meaningful units in the interviews were identified for different phases in palliative care, guided by Thompson's theory of involvement (Thompson, 2007; Thompson, Ruusuvuori, Britten, & Collins, 2007). By using a narrative approach (Holloway & Freshwater, 2007), we coded palliative care into an early palliative phase, a middle palliative phase, a terminal phase and a bereavement phase. We defined the early palliative phase as the first days following the diagnosis of incurable disease, the middle palliative phase as the time between the early phase and the terminal phase, the terminal phase as the last weeks before death and the bereavement phase as the period following after the patient's death. By focusing on content, form and context in the storyline of the interviewees, a theme was identified for each phase and the story was subsequently organized with a chronological structure (Patton, 2015). The themes were built by organizing the data into increasingly more abstract units (Table 3). The inductive process involved working back and forth between the data and the themes until the researchers had a comprehensive understanding of the interviews (Creswell, 2014). The researchers worked together in the analysis process and various interpretations were useful for developing an intersubjective understanding of the narratives (Wertz et al., 2011).

**Table 3:** Illustration of analytic steps followed to identify relevant themes

Coding	Quotations	Subtheme	Theme
Early palliative phase	“We felt well informed; The doctor told it like it was”	Information‐giving	Limited involvement in the early phase
	“We respected her desire not to be too informed about prognosis, but I would have liked to know a bit more”	Family caregivers' independent need for information	
	“He decided. He let us know early on that he wanted to stay at home”	The patient decided	

### 2.7 Rigour

The informants' stories are interpreted and retold by researchers and researchers can be seen as co‐authors of the narratives in a study (Holloway & Freshwater, 2007). However, credibility is a fundamental goal of qualitative research (Holloway & Freshwater, 2007; Polit & Beck, 2012).

The first author had experience as an oncology nurse. Being that close to the field could represent an obstacle to an open‐minded and impartial position (Patton, 2015). The interviewer and the interviewees did not know each other. To prevent bias, the co‐authors were strongly involved in the analysis through the process of communicative validation (Brinkmann & Kvale, 2015). All the authors read the transcripts and participated in discussions about coding and identifying the themes. The themes derived from the data (Tong, Sainsbury, & Craig, 2007) expressed involvement in different phases of palliative care. The findings include rich descriptions to increase transferability (Polit & Beck, 2012).

## 3 FINDINGS

Eleven family caregivers shared their experiences being involved in palliative care. The narratives consisted of four interrelated themes: (a) limited involvement in the early phase; (b) emphasis on patient‐centred care in the middle phase; (c) lack of preparation for the dying phase; and (d) lack of systematic follow‐up after death. Low involvement was a common feature in the stories.

### 3.1 Limited involvement in the early phase

In the early phase, the family caregivers felt that they were thoroughly informed about the diagnosis, treatment and severity of the disease: A female spouse told it like this: “We felt well informed; The doctor told it like it was” (FC‐7). The message that the patient had entered the palliative phase was generally provided by a physician at the hospital. In most of these situations, the family caregivers were present when the information was given. However, one of the informants felt that the seriousness of the diagnosis was excessively stressed. Another emphasized the importance of information to minimize uncertainty regarding palliative care for the patient and the family caregivers. “I would have liked a bit more information, not necessarily about when it would be over, but about how the process would be” (FC‐10). This young husband also said that the information provided depended on the question he asked.

However, despite being satisfied with the thoroughness of the information provided, several family caregivers expressed a desire to speak with health personnel about the expected disease trajectory and how this would affect their role as caregivers and the family situation. They expressed that they wanted more information about how the disease would develop and what to expect in the different phases of the illness. Importantly, the caregivers' and patients' desires for information were not always congruent. One son of a mother expressed this: “We respected her desire not to be too informed about the prognosis, but I would have liked to know a bit more” (FC‐5). One family caregiver wished to speak with the physician without the patient present. However, the family caregiver found it difficult to make this request in the presence of the patient. Another family caregiver was invited to speak with the physician but found it difficult to accept the offer in front of the patient. A young husband expressed: “That would mean you want to talk about something that you can't address with the patient present” (FC‐10). These difficulties hindered the family caregivers from having a dialogue with healthcare providers and from obtaining the information they needed to be prepared for the different phases of the palliative care.

Although the patient wished to die at home, several family caregivers expressed ambivalence in this regard. A female spouse said: “He decided. He let us know early on that he wanted to stay at home” (FC‐3). These family caregivers felt that they were not part of that decision and some felt it was difficult to fulfil the patient's wish. The family caregivers expressed that they were offered few opportunities to define their own needs and challenges. “I was there to care for him and look after him—my own needs were neglected” (FC‐3).

Some of the family caregivers had expressed to healthcare providers at an early stage that they did not want the patient to die at home because they did not feel able to bear the burden. “They wanted us to care for our mother at home, but we could not take that responsibility” (FC‐8). In cases such as this, the patients spent the terminal phase in a nursing home.

### 3.2 Emphasis on patient‐centred care in the middle phase

In the middle phase of palliative care, the family caregivers noted that the patients' wishes and needs were taken seriously by the healthcare providers both in the hospital and in primary care. However, they expressed that their own needs as family caregivers were occasionally neglected. A female spouse said that although she felt she was listened to, only her husband's illness and needs were discussed. She had told the healthcare provider that caring for her husband was too burdensome for her. “I told the nurses, but it was my husband who decided” (FC‐4). Another female spouse described this issue as follows: “Obviously, we were given the opportunity to discuss our problems, but it depended on what he would accept” (FC‐6). In some cases, the patient's unwillingness to accept help prevented family caregivers from enlisting necessary aid from healthcare services. “It was extremely tiring, because he didn't want me to bring in a lot of people and make such a fuss; I was supposed to take care of everything and be in control all the time” (FC‐3). The role of caregiver overshadowed the role of family member and prevented caregivers from being close to the patient and providing emotional support. A wife expressed the following: “When he was ill with vomit and diarrhoea, I cared for him, but in a way I couldn't be close to him, near him; I was an assistant. I wished I had more time with him” (FC‐6). The family caregivers often felt they had too much responsibility.

Several said that they did not use the offered services enough, or that they used them too late because they were unable to foresee what kind of services they would need. A female spouse said: “I should have asked for help much earlier. When I got help, I hadn't slept for three months” (FC‐3). She acknowledged that her husband's unwillingness to receive health services in their home was a reason for the delay.

While the patient was in a primary healthcare setting, the family caregivers were uncertain about who was responsible for medical treatment. A female spouse expressed this: “I thought it was the physician on the palliative team or in the cancer unit who was in charge and not the family doctor” (FC‐11). It was confusing to not understand who was in charge or who the caregivers should contact when a need arose. The informants wanted the family doctor to have a central part in palliative care and some said that the family doctor had provided good support and information. A son expressed it like this: “The family doctor communicated well with other health personnel who were involved in the treatment. Thus, we got important information that mother had short time to live” (FC‐5).

A wife expressed that the nurse wanted the family doctor to pay the patient a visit at home to assess the situation because the patient's condition was deteriorating. However, the patient did not want the doctor to visit: “I suspected that he was afraid the family doctor would send him to the nursing home” (FC‐6). This family caregiver wanted the family doctor to be involved in palliative care. The family caregivers expressed that there were conflicts of interest between patients and family members regarding various decisions in palliative care.

### 3.3 Lack of preparation for the dying phase

The family caregivers experienced a lack of involvement in planning for the terminal phase. While they felt a heavy responsibility, none talked about being involved in making plans for the terminal phase. The family caregivers experienced this phase as difficult, as they had no knowledge about what lay ahead. A son put it like this: “I wondered whether she would be in a coma for days” (FC‐5). A female spouse of a patient who had experienced a difficult course of illness said that if she had known how challenging providing care in the terminal phase would be, she would not have taken on the burden. The need for more information about the process of dying was therefore emphasized by several family caregivers.

The family caregivers said that they wished they had contacted health personnel at an earlier stage in palliative care, as it would have enabled them to obtain necessary help and information when they needed it. “There was no contact until I contacted them” (FC‐3). The informants described how important it was to have someone from healthcare services to call if needed. Among the family caregivers, establishing a dialogue with healthcare providers and being listened to were considered important steps to improving their involvement in the terminal phase. However, some experienced situations where the patient was unwilling to provide information about his/her condition to family caregivers, preventing cooperation between health personnel and family caregivers.

When the patients were at home, the caregivers often experienced being alone with the responsibility for the patient's care. The burden of providing care in the final stages of life was not recognized by healthcare professionals, and the family caregivers sometimes felt they were left to handle the tasks on their own. Taking responsibility for her husband both night and day was perceived as frightening and challenging. Conversely, when visiting the patient in a nursing home, they felt well cared for by the health professionals. A female spouse told about the experience of visiting the patient. “It was good to be at the nursing home. The girls and I, we were all there and we were treated in a nice way” (FC2).

### 3.4 Lack of systematic follow‐up after death

The family caregivers shared different stories about their experience with follow‐up after the death of the patient. Some met with the local community oncology nurse and some met with healthcare providers from the hospital. The informants believed there should be an offer of follow‐up, even though some declined such an offer. One female spouse stated the need for a systematic follow‐up offer after death: “I think it should have been a systematic process regarding this” (FC‐6). The family caregivers expressed that it was especially important to talk with the nurse who had been present on the last day of the patient's life, as they had many questions about the process of dying. “There are many questions I would like to ask about what happened in the last hours he lived” (FC‐1). Such answers were considered important in the process of grieving and moving on with their lives.

The family caregivers experienced a lack of support in the bereavement process. Most of the informants expressed that there had been no offer of follow‐up. A female spouse said it like this: “Maybe some more contact afterwards. Questions about me and how I was doing” (FC‐1). This family caregiver expressed that she had received support from the local priest and her own family doctor. Among the caregivers, being contacted by health professionals after the patient died was seen as important to be able to process their sorrow and move on with life. Based on the stories told, there seemed to be a lack of systematic follow‐up from healthcare providers. The family caregivers expressed that a conversation after the patient's death might have been valuable in helping to get over the sorrow and loss.

## 4 DISCUSSION

The aim of this study was to obtain insight into how family caregivers experience involvement in palliative care. The family caregivers expressed that although they were well informed about the patients' diagnoses, they experienced low levels of involvement in defining problems and challenges regarding the care of the patient. While the informants felt that the patient received patient‐centred care, they felt that their own needs were neglected. They also felt unprepared for the process of dying. The family caregivers experienced a lack of systematic follow‐up after the patient died. Dialogue and being listened to by the healthcare providers were highlighted as important.

While the family caregivers were well informed about the patients' diagnoses, they also wanted to be more involved in the decision‐making process regarding palliative care. It appeared that the need for information was mostly defined by the patient and healthcare providers and that their communication was characterized by paternalism (Thompson et al., 2007). Thompson et al.'s (2007) components of participation were not readily identified in the informants' descriptions of involvement, and this was exacerbated when the symptom burden was high and when the patient did not want to receive appropriate health services. The framework for involvement and participation described by Thompson et al. (2007) was useful in highlighting shortcomings in the involvement of family caregivers. This holistic framework consists of three elements: components, levels and context, which were valuable in identifying family caregivers' involvement in different phases of palliative care.

In the present study, the family caregivers wanted more information about practical issues related to the daily care of the patient. Funk et al. (2010) suggested that being involved in care and feeling able to effectively provide palliative care can strengthen family caregivers. The lack of preparation, knowledge and ability of family caregivers is well known. Earlier research has shown that the feeling of being unprepared, especially regarding knowledge of symptoms and decisions about medication management, can represent a considerable burden for many family caregivers (Funk et al., 2010; Rakic et al., 2018). The family caregivers in our study shared stories indicating that the dying person and his/her caregivers have different needs, and this is well known in the literature (Male, Fergus, & Stephen, 2015; Pottle et al., 2017). The need for information and the provision of practical support are two areas where patients' and family caregivers' opinions can differ. The rhetoric around patient‐centred care (Etkind et al., 2015; Kitson et al., 2013; Robinson et al., 2008) may prevent awareness of family caregivers' unmet needs in palliative care. Hence, a family perspective should be included in the concept of patient‐centred care.

In our study, the caregivers talked about how difficult it was to be in the role of an “assistant” instead of the role of a close family member. Studies have reported that changing roles can be difficult for family caregivers involved in palliative care (Fringer et al., 2018). To handle multiple roles could lead to over‐exertion and these contradictory roles must be balanced (Fringer et al., 2018). In our study, this need for balance was significant, especially when the patient rejected support offered by healthcare providers. Research has showed the importance of a personal relationship between family caregivers and health personal and detailed information about the dying process to handle the challenges (Mohammed at al., 2018; Røen et al., 2018).

We found that healthcare providers are not always sufficiently aware of family caregivers' needs for information and practical support, especially in the terminal phase. From an ethical perspective, it has been suggested that family caregivers should be conceptualized as an integral component of the patient's identity and should therefore be included in the decision‐making process from the beginning rather than being seen as a third party to the doctor–patient relationship (van Nistelrooij et al., 2017). Given the crucial role of family caregivers in providing home‐based care, maintaining a high degree of involvement and support for family caregivers in palliative care is warranted.

Our informants experienced the terminal phase as being particularly difficult. A lack of knowledge about the last phase left them poorly prepared for what lay ahead. Previous research has shown that health professionals underestimate family caregivers' need for information about palliative care, death and dying (Collins, McLachlan, & Philip, 2018). Family caregivers need healthcare providers to explain in a clear language what is going to happen during the process of dying, without assuming any prior understanding (Dose et al., 2015).

The family caregivers also experienced a lack of follow‐up after the patient had died. Both the specialist health service and the municipal health service had routines in relation to following up with bereaved individuals. Continued follow‐up of family caregivers is part of the guidelines from the WHO for palliative care (Integrating Palliative Care & Symptom Relief into Primary Health Care, 2018), but it appears that these guidelines may not be manifested in a systematic way. Implementing assessment tools to evaluate needs for support might raise awareness among healthcare providers about the needs of family caregivers, including caregivers' obvious needs and needs they are unaware of (Ewing & Grande, 2013).

The findings from this study show deficiencies in the involvement of family caregivers in various phases of palliative care. Family caregivers' narratives can be used to improve various assessment tools that might strengthen their involvement in palliative care.

### 4.1 Limitations

The informants might be grieving when they were interviewed, and this could have influenced the way they communicated their experiences. The interviews were conducted 3–12 month after the family member's death. This might have affected how family caregivers remembered what had happened. The findings represent the family caregivers' subjective experiences as interpreted by the researchers. The findings from our study cannot be generalized, but it is reasonable to assume that the findings can be applied to similar situations and contexts as well as being family caregivers to patients with other diagnoses.

## 5 CONCLUSION

Health authorities recommend that patients receiving palliative care should have the opportunity to spend more time at home at the end of their lives. This entails increased responsibility for family caregivers. This study demonstrated that family caregivers experience limited of involvement in planning palliative care. Their voices seem to be silent and the involvement of family caregivers is not in proportion to their responsibilities. Consequently, the needs of family caregivers in the palliative care trajectory must be addressed to successfully provide home‐based care. Family caregivers' involvement in palliative care should be a topic in nursing education and continuing education for nurses.

## CONFLICT OF INTEREST

The authors did not declare any conflict of interests.

## AUTHOR CONTRIBUTIONS

AST, MK, BJL and TH: Made substantial contributions to conception and design, or acquisition of data, or analysis and interpretation of data. AST, MK, BJL, TH, MT and TSM: Involved in drafting the manuscript or revising it critically for important intellectual content. AST, MK, BJL, TH, MT and TSM: Given final approval of the version to be published. Each author should have participated sufficiently in the work to take public responsibility for appropriate portions of the content. AST, MK, BJL, TH, MT and TSM: Agreed to be accountable for all aspects of the work in ensuring that questions related to the accuracy or integrity of any part of the work are appropriately investigated and resolved.
